# Responsiveness and minimal important change of the QuickDASH and PSFS when used among patients with shoulder pain

**DOI:** 10.1186/s12891-020-03289-z

**Published:** 2020-05-27

**Authors:** Tarjei Rysstad, Margreth Grotle, Lars Petter Klokk, Anne Therese Tveter

**Affiliations:** 1 Faculty of Health Sciences, Department of Physiotherapy, Oslo Metropolitan University, P.O. Box 4, St Olavs Plass, Oslo, Norway; 2 Research and Communication Unit, Oslo University Hospital, Oslo, Norway; 3 Multidisciplinary outpatient clinic, Department of physical medicine and rehabilitation, Ålesund hospital, Ålesund, Norway

**Keywords:** Clinimetrics, Outcome measures, Functional limitation, COSMIN

## Abstract

**Background:**

The Quick Disabilities of the Arm, Shoulder and Hand questionnaire (QuickDASH) and the Patient-Specific Functional Scale (PSFS) are commonly used outcome instruments for measuring self-reported disability in patients with shoulder pain. To date, few studies have evaluated the responsiveness and estimated their minimal important change (MIC). Further assessment will expand the current knowledge and improve the interpretability of these instruments in clinical and research practice. The purpose of this prospective cohort study with 3 months follow-up was to evaluate the responsiveness of the QuickDASH and PSFS in patients with shoulder pain, and to estimate their MICs by using two different anchor-based methods.

**Methods:**

Patients with shoulder pain recruited at a multidisciplinary hospital outpatient clinic completed the QuickDASH and PSFS at baseline and at 3 months follow-up. The responsiveness was evaluated by using a criterion approach with the area under the receiver operating characteristic curve (AUC) and a construct approach by testing 9 a-priori hypotheses. The MIC was assessed using two anchor-based MIC methods.

**Results:**

134 patients participated at baseline and 117 (87.3%) at 3 months follow-up. The AUC was acceptable for both QuickDASH (0.75) and PSFS (0.75). QuickDASH met 7 (77.8%) and PSFS 8 (88.9%) of the hypotheses. None of the instruments showed signs of floor and ceiling effects. The MIC estimates ranged from 10.8 to 13.6 for QuickDASH and from 1.9 to 2.0 for PSFS, depending on the method used.

**Conclusion:**

This study demonstrates that both the QuickDASH and PSFS are responsive measures of disability in patients with shoulder pain. The estimated MIC values were presented.

## Background

Shoulder pain is a common musculoskeletal condition that can often lead to considerable disability [1], impacting the performance of daily activities and restrict participation in major life areas such as work, education, community, social and civil life [2, 3].

It is important to capture the patients’ functional disability in both clinical practice and research. Patient-reported outcome measures (PROMs) can be used to assess patients’ perceived degree of disability at both individual and group level. In the last decade, several region-specific and patient-specific questionnaires for assessing functional status in shoulder patients have been developed [4–6]. Of these, the Disabilities of the Arm, Shoulder and Hand questionnaire (DASH) and its short version (QuickDASH) are frequently used PROM and their measurement properties have been widely evaluated in patients with proximal upper extremity disorders [7–12]. A recent systematic review highlighted the scant evidence investigating the measurement properties of the QuickDASH in patients with shoulder pain [13]. Another questionnaire that has received considerable attention is the Patient-Specific Functional Scale (PSFS), which is eliciting activities that are most important to the individual patient. Several guidelines have recommended the use of the PSFS in management of different musculoskeletal conditions [14–16]. Others have also recommended the use of the PSFS in addition to condition-specific measures to complement the activity and participation components [6, 17]. If used as PROMs in clinical or research settings, high-quality studies to thoroughly evaluate their measurement properties are required [18].

The measurement properties of a PROM are population and context-specific, and should therefore be evaluated in different patient populations and clinical study contexts before they are used in clinical and research practice [19, 20]. In addition to reliability and validity, responsiveness is an important measurement property which aims to evaluate the PROMs ability to capture change over time [21]. Ideally, the responsiveness of an instrument used as an outcome in clinical or research environments should be high [20].

For the interpretation of change scores among individual patients, the Minimal Important Change (MIC) is an important estimate for both clinicians and researchers who are using the PROM. The MIC is defined as ‘the smallest change in score that patients perceive to be important’ [20], where a change score exceeding this value would provide information to the clinician that a change in treatment has occurred [19]. A number of anchor-based and distribution-based methods have been used to determine the MIC [19, 22, 23]. The COSMIN group recommends anchor-based methods for estimating the MIC because they relate to an external anchor regarding the patient’s perceived change of the treatment [20]. It has been recommended that researches use multiple methods to triangulate MIC results because the MIC is not a fixed value but influenced by context, calculation method and baseline severity [19, 22, 24]. Recently, a predictive modelling MIC method has been found to be a more accurate calculation of the anchor-based MIC [23, 25]. However, this method has never been used to calculate the MIC for PROMs used in patients with shoulder pain. Moreover, the responsiveness and MIC values of the Norwegian versions of the QuickDASH and PSFS has not been assessed before in a cohort of patients with shoulder pain undergoing physical therapy.

This study aims to expand on this current knowledge by evaluating the responsiveness and the MIC of both the QuickDASH and PSFS in patients with shoulder pain.

## Methods

### Study design

This study is a prospective cohort study with 3-months follow-up. Outcomes were measured at baseline and 3 months after undergoing physiotherapy treatment. Ethical committee approval was obtained from the local ethical committee (2018/1191 C). All participants signed informed consent.

### Participants

Participants were recruited from a multidisciplinary hospital outpatient clinic for shoulder patients at Ålesund Hospital in Norway between March 2015 to January 2018. All potential participants received a detailed explanation of the study from the research coordinator. Participants were eligible for inclusion if they were diagnosed with shoulder pain by one of the physicians at the clinic, aged 18 years or older, and adequately understood the Norwegian language. Exclusion criteria were systematic disease or generalised pain, cardiac disease, symptoms of cervical spine disease or surgery in the affected shoulder within the last 6 months.

### Treatment

The patients were referred to ‘usual physiotherapy treatment’ for the management of their shoulder pain within primary and secondary care. The physiotherapy sessions were not standardised when it comes to how many treatment sessions were given, length and components of the intervention.

### Outcome measures

At baseline, all included patients completed a booklet of questions (paper and pen administered) prior to their first consultation with the physician at the outpatient clinic. The booklet consisted of demographic variables, QuickDASH, PSFS and comparator instruments, all in Norwegian. At the 3-month follow-up, the participants were also requested to fill out a Global Rating of Change scale in addition to the baseline questions.

#### QuickDASH

The QuickDASH consists of 11 questions covering 6 domains (daily activities, symptoms, social function, work function, sleep, and confidence) [26]. Each item is rated on a 5-point Likert scale, from 1 (no difficulty) to 5 (unable). The score is converted into a 100-point scale, where 100 represents greatest disability. Ten of 11 items are necessary for calculating the QuickDASH score [9, 10]. A strong correlation has been found between the QuickDASH and its longer version (DASH) [10, 27], and support for both these questionnaires in shoulder patients has been reported recently [4, 11, 28, 29]. The MIC has been reported in patients with upper extremity conditions, ranging from 8.2 to 13.4 [30–32]. The cross-cultural adapted Norwegian version was used in this study [33].

#### The Patient-Specific Functional Scale

The PSFS consists of a standardised script for eliciting activities that are most important to an individual patient [34]. In the original version of the PSFS, patients are asked to define 3 to 5 activities they are having difficulty with. In this study, patients were asked to define 3 main activities currently difficult or impossible to perform as a result of their condition or injury. Of these 3 activities, the patients were asked to identify the most important one. Each activity was rated on an 11-point scale, 0–10, where 0 is “Unable to perform the activity” and 10 is “Able to perform the activity at the same level as before injury or problem”. An average PSFS score was obtained by summing the ratings of the nominated activities and dividing by the number of defined activities (up to 3). Studies have supported the use of PSFS in patients with shoulder pain [5, 6, 35]. The MIC has also been reported in patients with upper extremity conditions, ranging from 1.4 to 2.7 [36, 37].

#### Comparator instruments

Pain intensity was measured asking patients to rate their average shoulder pain over the last 2 weeks on a *Numeric Rating Scale (NRS)*, ranging from 0 (´no pain´) to 10 (´the worst imaginable pain´). The NRS has shown to have good validity and responsiveness in patients with shoulder disorders [35]. *Workability* was measured by the single item “Current workability compared with the lifetime best” from the Work Ability Index (WAI), scores range from 0 to 10, higher score indicates better work ability [38]. Kinesiophobia was measured with a single question, referred to as the single *Substitute Question of Kinesiophobia (SQK)*: “How much ‘fear’ do you have that these complaints would be increased by physical activity?”, scores range from 0 to 10, where higher score indicates more kinesiophobia [39, 40]. Emotional distress was measured with the *Hopkins Symptom Checklist (HSCL-25)*, consisting of 25 items that are rated from 1 (´not at all´) to 4 (´extremely´). The total score, average of the 25 items, was calculated [41].

#### Global Perceived Effect scale (GROC)

At the 3 months follow-up, the participants also completed a global rating of change scale (GROC) and were asked to rate their change in shoulder function in relation to the most important activity (“Compared to the start of the treatment and related to my most important activity rated in the PSFS, I am now feeling:”) on a 7-point Likert scale with the response categories: (1) very much improved, (2) much improved, (3) slightly improved, (4) unchanged, (5) slightly worsened, (6) much worsened, and (7) very much worsened. Different GROC scales have shown good test-retest reliability in several musculoskeletal disorders, including shoulder pain [42].

### Statistical analysis

All statistical analyses were performed with SPSS version 24 for Mac (IBM Corporation, Armonk, NY). Descriptive statistics were computed to describe the sociodemographic and clinical characteristics. Change scores of the QuickDASH, PSFS and comparator instruments were obtained by subtracting the follow-up score (3 months) from the baseline score. Data were considered incomplete if more than 2 items of the QuickDASH were missing, if none activities were reported in the PSFS, or the GROC score was missing. These incomplete data were not included in the data analysis. For both responsiveness and MIC assessment, Cohen’s correlation threshold of 0.35 was used to define an acceptable association between the anchor (GROC) and the PROMs change scores [19, 43].

This study followed the recommendation of the COSMIN group [20] and the COSMIN Risk of Bias checklist [18] when determining responsiveness and MIC of the QuickDASH and PSFS.

#### Floor and ceiling effects

The presence of floor or ceiling effects has a consequence for the responsiveness and MIC of a PROM, since the patients cannot show any further change. Floor or ceiling effects were considered to be present if more than 15% of the respondents achieved the minimum or maximum of possible score [20].

#### Responsiveness assessment

Responsiveness was, according to the COSMIN guidelines [21, 44], assessed by 2 methods: (1) the criterion approach by assessing the area (AUC) under the Receiver Operating Curve (ROC) and (2) the construct approach by hypotheses testing.

To assess the criterion approach, the population was dichotomised into an ‘improved’ group and an ‘unchanged’ group. There is no consensus of the categorisation of the GROC concerning the improved and unchanged group, and various categories have been used [31, 45, 46]. In this study, patients classified as ‘very much improved’ and ‘much improved’ on the GROC were considered improved, and those classified as ‘slightly improved’, unchanged’ and ‘slightly worsened’ were considered unchanged [20]. Slight changes are therefore considered as less likely to be clinically meaningful. Patients who reported deterioration were excluded. The AUC was calculated as the ability of the QuickDASH and PSFS to discriminate between patients classified as ‘improved’ and ‘unchanged’. An AUC of at least 0.70 was regarded as acceptable responsiveness [20].

To assess the construct approach, 9 a-priori hypotheses were formulated and tested for both the QuickDASH and PSFS. These hypotheses were based on reported evidence about the PROMs and consensus among the study investigators, described in Table 1. The data were assumed to be normally distributed if there was no or minimal difference between the mean and median value, confirmed by histograms, Q plot and the Shapiro-Wilk test. Pearson correlation coefficient was used if the data were normally distributed, otherwise, a Spearman’s rank correlation coefficient was used. A correlation of less than 0.30 indicates a weak correlation, at least 0.30 and less than 0.60 indicates moderate correlation, and a correlation at least 0.60 indicates good correlation [53]. The standardised response mean (SRM) was calculated by dividing the mean change score by the standard deviation (SD) of the change. The effect size (ES) was calculated by dividing the mean change score by the SD of the baseline scores [54]. An instrument was considered having acceptable responsiveness, based on the construct approach, if meeting at least 75% of the hypotheses according to the COSMIN guidelines [20].

**Table 1:** Predetermined hypotheses for evaluating the responsiveness of the QuickDASH and PSFS

**Hypotheses**		**QuickDASH**	**PSFS**
**1**	The correlation between the QuickDASH/PSFS change score and the GROC is negative and moderate.	+	+
**2**	The ES and SRM of the QuickDASH/PSFS are < 0.2 for patients classifying themselves as ‘unchanged’ on the GROC.	+	–
**3**	The ES and SRM of the QuickDASH/PSFS are ≥0.5 for patients classifying themselves as ‘much improved’ on the GROC.	+	+
**4**	The correlation between the QuickDASH change score and the PSFS change score is moderate (> 0.30 and < 0.60). Since both these PROMs measure the same construct (i.e. disability/function), we expected the magnitude of this correlation to be moderate.	+	+
**5**	The correlation between the QuickDASH/PSFS change score and the NRS change score is moderate (> 0.30 and < 0.60). This hypothesis is based on the following research literature showing that PSFS correlates moderately with the NRS in upper extremity patients [5, 36].	–	+
**6**	The correlation between the NRS and QuickDASH change score is higher (at least 0.1) than the correlation between the NRS and PSFS change score. Based on a recent study [35], and the understanding that the QuickDASH emphasise the construct of pain higher than the PSFS, we expected it to correlate higher with the NRS.	+	+
**7**	The correlation between the QuickDASH/PSFS change score and the SQK change score is moderate (> 0.30 and < 0.60). This hypothesis is based on previous studies showing that fear of movement scales correlates moderately with shoulder disability scores [47, 48].	+	+
**8**	The correlation between the WA and PSFS change score is higher than the correlation between the WA and QuickDASH change score. Recent studies show that shoulder patients report work/employment as PSFS items [6, 17]. The QuickDASH disability questionnaire used in this study does not capture work in a direct way [10]. Therefore, we expected lower correlation between the WA and QuickDASH compared to the correlation of WA and PSFS.	+	+
**9**	The correlation between the QuickDASH/PSFS change score and the HSCL-25 change score is low (< 0.30). This rationale is based on previous studies showing that QuickDASH and PSFS correlates low with mental health component scores [49–51]. The Norwegian version of HSCL-25 has shown strong correlation with mental health scores [52].	–	+
	n (%)	7/9 (77.8)	8/9 (88.9)

Abbreviations: – = unmet hypothesis; / = not applicable hypothesis; + = met hypothesis; ES = effect size; GROC = global rating of change scale; HSCL-25 = Hopkins Symptom Checklist; NRS = numeric pain rating scale; PSFS = Patient-Specific Functional scale; SRM, standardised response mean; SQK = Single substitute question for kinesiophobia; QuickDASH = Quick Disabilities of the Arm, Hand and Shoulder questionnaire; WA = Workability

#### MIC assessment

The MICs were calculated with 2 anchor-based methods for MIC estimation; the ROC method (MIC_ROC_) and the predictive modelling method (MIC_pred_). The GROC was used as an anchor in both methods. When it comes to the estimation of the PSFS MIC, the PSFS scale was reversed.

To assess the MIC_ROC_, the anchor distinguishes between patients who are ‘improved’ and patients who are considered ‘unchanged’, based on the same criteria as the responsiveness assessment. The MIC was estimated as the optimal cut-off point on the ROC curve, the value that represents the lowest overall misclassifications where both sensitivity and 1-specificity are maximised [19, 20]. The sensitivity relates to the proportion of improved patients according to the anchor who is correctly classified as improved by the PROM. The specificity is the proportion of unchanged patients according to the anchor who is correctly identified by the PROM as not changed.

The MIC_pred_ is based on a logistic regression, using the dichotomised anchor response to predict whether a patient belongs to the improved or unchanged group using the change in the QuickDASH/PSFS scores as the predictor [25]. The MIC_pred_ is calculated using the equation [ln(pre-odds) – C]/B, where C is the intercept and B is the regression coefficient for the change in the QuickDASH/PSFS scores from the logistic regression model [25]*.* If the proportion of improved participants on the GROC is considerably smaller or larger than 0.50, it is suggested that an adjusted MIC needs to be calculated [23]. For the present study, the proportion of improved participants on the GROC was 0.48, therefore, an adjusted MIC was not calculated.

Since the MIC has shown to be influenced by the baseline score of the patients [24], we carried out a subgroup analysis to assess the difference in MIC values with high and low baseline QuickDASH/PSFS scores. The median QuickDASH/PSFS baseline score was used to divide the population into the two subgroups. The ROC method (MIC_ROC_) was used when estimating the MIC for baseline scores.

## Results

A total of 241 patients with shoulder conditions were referred to the hospital-based outpatient clinic and invited to participate in the study. One hundred and thirty-four patients met the inclusion criteria, accepted the invitation and were recruited for the study. Of these patients, 17 did not complete the follow-up assessment at 3 months. In total, 117 patients (87.3% of the baseline population) were included in the analysis of the construct approach of responsiveness. Of these, 11 patients were excluded due to the missing-item criterion, resulting in 106 patients (79.1% of the baseline population) included in the analysis of the criterion approach of responsiveness and MIC estimation. Baseline sociodemographic and clinical characteristics of the included patients are presented in Table 2. Ceiling and floor effects were not present in neither the QuickDASH nor PSFS.

**Table 2:** Baseline characteristics (*n* = 134)

**Variable**	**Value**^**a**^
Gender	
Male	36 (26.9)
Female	97 (72.4)
Age, mean (SD)	45.8 (10.5)
Language	
Norwegian	123 (91.8)
Other	10 (7.5)
Occupational status	
Employed	110 (82.1)
Unemployed	1 (0.7)
Student	4 (3.0)
Retired	3 (2.2)
Disability pension	5 (3.7)
On sick leave	56 (41.8)
Pain duration	
1–3 months	3 (2.2)
4–12 months	35 (26.1)
More than 12 months	93 (69.4)
QuickDASH 0–100, mean (SD)	37.3 (16.1)
PSFS 0–10, mean (SD)	
Activity 1	3.1 (2.0)
Activity 2	3.3 (2.0)
Activity 3	3.4 (2.3)
Total	3.5 (1.8)
NRS 0–10, mean (SD)	5.3 (1.9)
SQK 0–10, mean (SD)	4.8 (3.2)
WA 0–10, mean (SD)	4.4 (2.5)
HSCL-25, 1–4, mean (SD)	1.6 (0.4)

Abbreviations: HSCL-25 = Hopkins Symptom Checklist total score; NRS = numeric pain rating scale; PSFS = Patient-Specific Functional scale, higher scores represent higher levels of function; QuickDASH = Quick Disabilities of the Arm, Hand and Shoulder questionnaire, higher scores represent higher levels of disability; SD = Standard deviation; SQK = Single substitute question for kinesiophobia; WA = Workability

^a^Values are n (%) unless stated otherwise

### Responsiveness

**Fig. 1:**
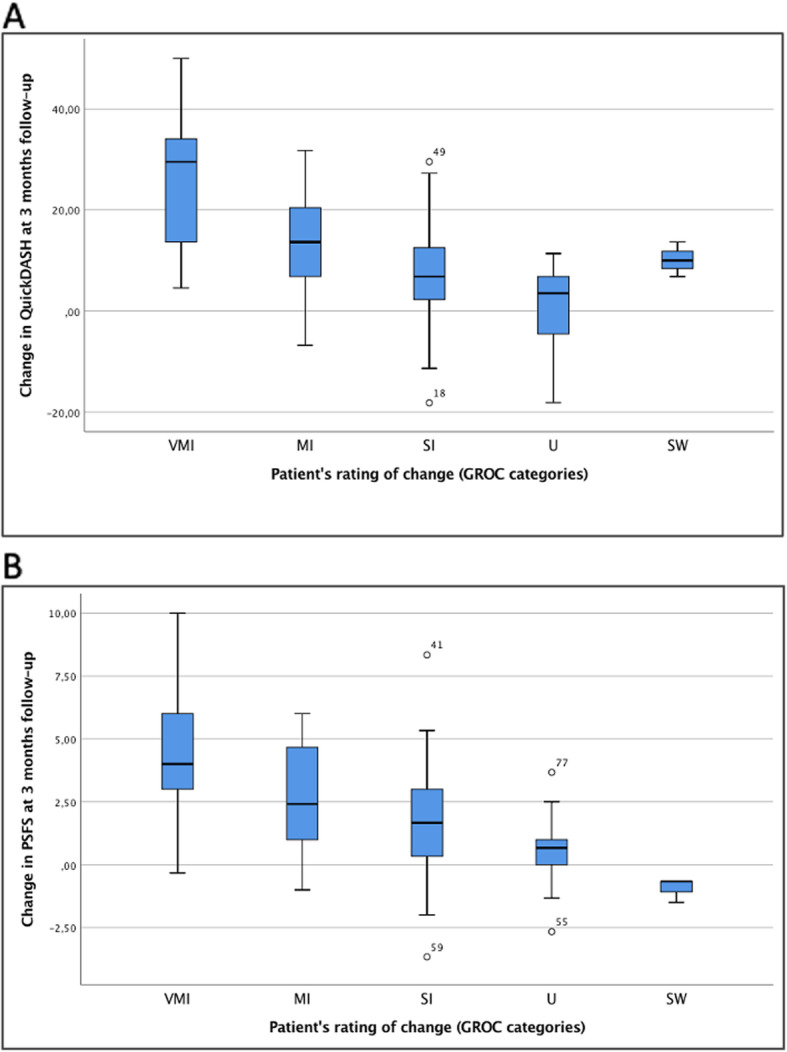
Box plots showing the distribution of the QuickDASH and PSFS change scores for the 7 GROC categories at 3-month follow-up. **a** QuickDASH change scores at follow-up, **b** PSFS change scores at follow-up. Abbreviations: GROC, global rating of change; MI, much improved; PSFS, Patient-Specific Functional Scale; QuickDASH, shortened version of the Disabilities of the Arm, Shoulder and Hand questionnaire; SI, slightly improved; SW, slightly worsened; U, Unchanged; VI, very improved; VMI, very much improved

The box plots in Fig. 1 show the distribution of the QuickDASH and PSFS change scores for each category of the GROC at the 3-month follow-up. There is considerable overlap between the distribution of scores for each category of the GROC for both questionnaires, except the ‘slightly worsened’-group (*n* = 3) of the PSFS change scores.

Table 3 presents scores, ESs and SRMs for QuickDASH and PSFS for the total sample, and subgroups classified according to each GROC category. In total, only 1 participant stated that he or she was much worse. None of the participants stated very much worse. For both the QuickDASH and PSFS, ESs and SRMs were progressively larger for each increment on the GROC, except for the 3 participants in the ‘slightly worsened group’. ESs and SRMs were large (> 0.9) for participants who were ‘very much improved’ or ‘much improved’ on the GROC. For the participants who stated ‘slightly improved’ on the GROC, the ES and SRM were moderate (0.4 and 0.6) for the QuickDASH and moderate to large (0.9 and 0.7) for the PSFS.

**Table 3:** Baseline, follow-up, change scores, effect size (ES) and standardised response mean (SRM) of the QuickDASH and PSFS according to the GROC category

**GROC category**	***N* (%)**	**Instrument**	B**aseline score, mean (SD)**	**Follow-up score, mean (SD)**	**Change score, mean (SD)**	**ES**	**SRM**
Total sample	106	QuickDASH PSFS	39.7 (17.2) 3.2 (1.7)	30.0 (18.7) 5.3 (2.4)	10.3 (13.1) − 2.1 (2.4)	0.6 1.2	0.8 0.9
Very much improved	14 (10.4)	QuickDASH PSFS	38.0 (16.8) 3.9 (2.0)	11.7 (11.7) 7.9 (2.2)	26.6 (15.4) −4.1 (2.6)	1.6 2.1	1.7 1.6
Much improved	36 (26.9)	QuickDASH PSFS	35.5 (14.4) 3.3 (1.6)	22.7 (13.2) 6.1 (2.0)	12.9 (11.2) −2.6 (2.0)	0.9 1.6	1.2 1.3
Slightly improved	34 (25.4)	QuickDASH PSFS	43.0 (17.3) 2.8 (1,8)	35.9 (17.5) 4.4 (1.6)	7.1 (11.1) −1.6 (2.4)	0.4 0.9	0.6 0.7
Unchanged	18 (13.4)	QuickDASH PSFS	48.1 (17.6) 2.9 (1.5)	47.2 (16.6) 3.5 (1.8)	0.9 (8.7) −0.6 (1.4)	0.1 0.4	0.1 0.4
Slightly worsened	3 (2.2)	QuickDASH PSFS	65.5 (16.6) 2.8 (1.1)	55.3 (13.3) 1.8 (1.4)	10.2 (3.4) 0.9 (0.5)	0.6 −0.8	3.0 −1.8
Much worsened	1 (0.7)	QuickDASH PSFS	- -	- -	- -	- -	- -
Very much worsened	0 (0.0)	QuickDASH PSFS	- -	- -	- -	- -	- -

Abbreviations: ES = effect size; GROC = global rating of change scale; NRS = numeric pain rating scale; PSFS = Patient-Specific Functional scale; SRM, standardised response mean; QuickDASH = Quick Disabilities of the Arm, Hand and Shoulder questionnaire

**Fig. 2:**
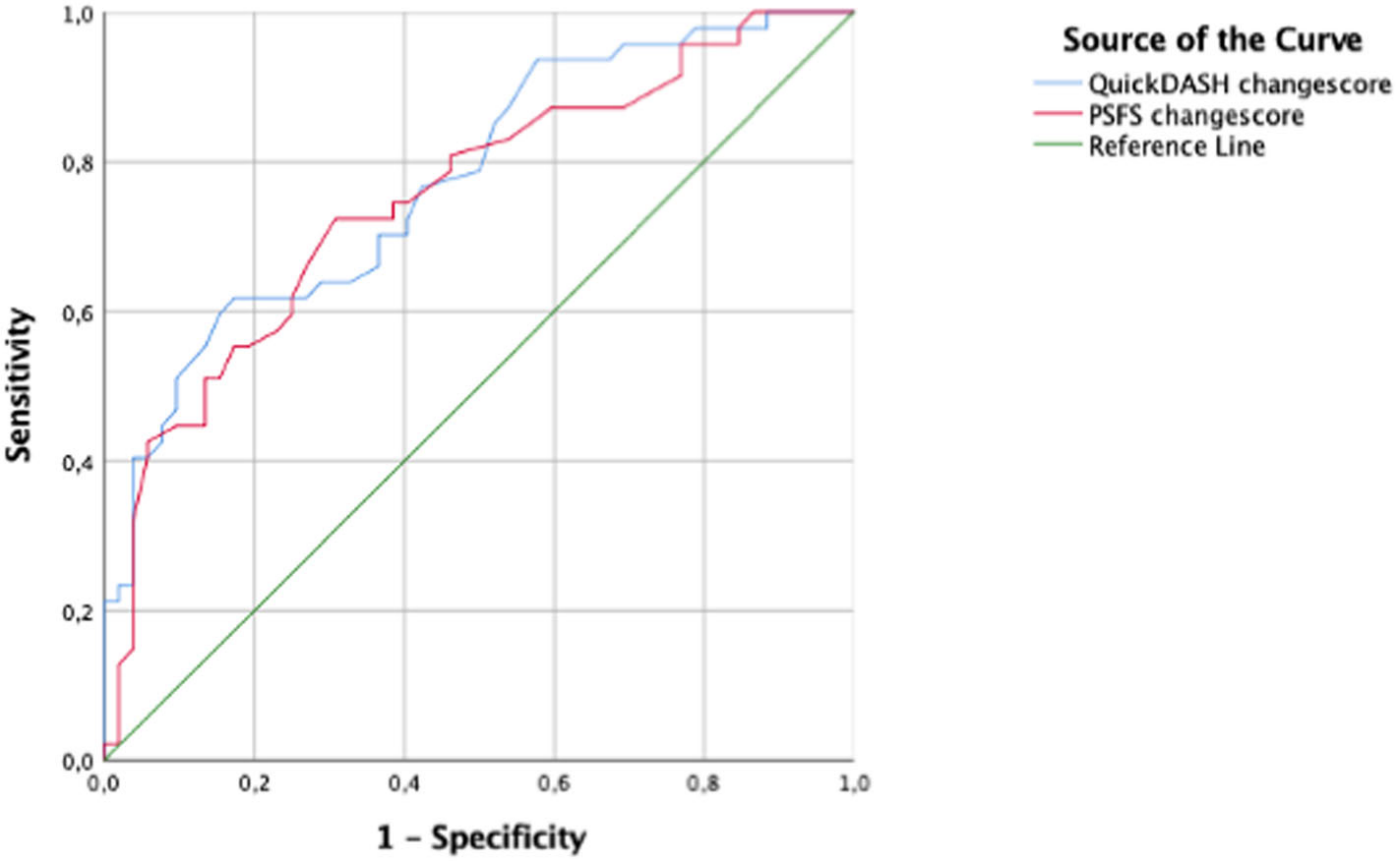
Receiver operating characteristic curves for the QuickDASH and PSFS for ‘improved’ and ‘unchanged’ on the GROC. QuickDASH at 3-month follow-up (area under curve = 0.75). PSFS at 3-month follow-up (area under curve = 0.75). Abbreviations: GROC, global rating of change; PSFS, Patient-Specific Functional Scale; QuickDASH, shortened version of the Disabilities of the Arm, Shoulder and Hand questionnaire

*Criterion approach of responsiveness*. Dichotomisation of the GROC showed that 50 patients (47.6%) improved and 55 patients (52.4%) were stable; 1 patient (0.9%) were excluded in the ROC curves analysis, since he or she had worsened clinical condition. The ROC curves (Fig. 2) were similar for both questionnaires, with an AUC for the QuickDASH of 0.75 (95% CI: 0.66, 0.84) and an AUC for the PSFS of 0.75 (95% CI: 0.65, 0.85). The responsiveness for both questionnaires was therefore considered satisfactory based on the criterion approach.

*Construct approach of responsiveness*. Responsiveness according to testing 9 a-priori hypotheses (Table 1) were met by both instruments; the QuickDASH met 7 hypotheses (77.8%) and the PSFS met 8 hypotheses (88.9%). The correlations between the QuickDASH/PSFS and comparator instruments are presented in Table 4.

**Table 4:** Correlations among the PROMs’ change scores (*n* = 117)

	**QuickDASH**^**a**^	**PSFS**^**a**^
**QuickDASH (0–100)**	–	0.45 (0.28, 0.59)
**PSFS (0–10)**	0.45 (0.28, 0.59)	–
**NRS (0–10)**	0.62 (0.49, 0.72)	0.32 (0.14, 0.49)
**SQK (0–10)**	0.37 (0.20, 0.52)	0.38 (0.20, 0.53)
**WA (0–10)**	0.44 (0.27, 0.58)	0.46 (0.29, 0.60)
**HSCL-25 (0–10)**	0.37 (0.20, 0.52)	0.25 (0.06, 0.42)
**GROC (1–7)**	−0.47 (− 0.61, − 0.31)	−0.50 (− 0.64, − 0.34)

Abbreviations: GROC = global rating of change scale; HSCL-25 = Hopkins Symptom Checklist total score; NRS = numeric pain rating scale; PSFS = Patient-Specific Functional scale; SQK = Single substitute question for kinesiophobia; QuickDASH = Quick Disabilities of the Arm, Hand and Shoulder questionnaire; WA = Workability

*n* refers to the total sample sizes and may deviate in some of the correlation analysis due to missing data

All correlations were significant at *P* < 0.01

^a^Values in parentheses are 95% confidence interval

### Minimal important change

**Fig. 3:**
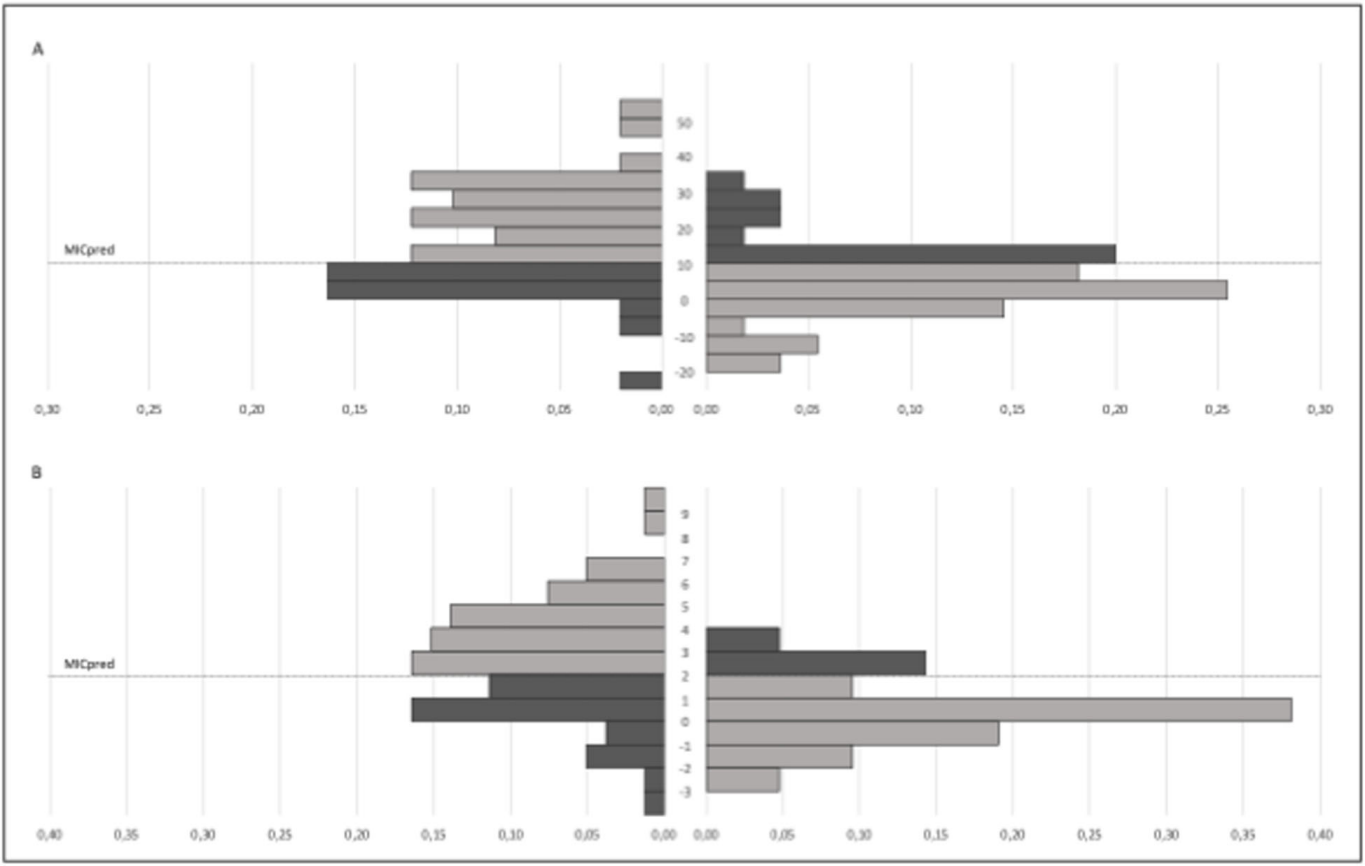
Visual anchor-based MIC distribution of 50 improved and 55 unchanged patients according to the anchor. **a** The vertical axis depicts the QuickDASH change score. The dotted line represents the MIC_pred_ value of 10.8. The light shaded patients are correctly classified, whereas the dark shaded patients are misclassified. 28.0 and 32.7% of the improved and unchanged patients were misclassified, respectively. **b** The vertical axis depicts the PSFS change score. The dotted line represents the MIC_pred_ value of 1.9. The light shaded patients are correctly classified, whereas the dark shaded patients are misclassified. 38.0 and 29.1% of the improved and unchanged patients were misclassified, respectively. Abbreviations: PSFS, Patient-Specific Functional Scale; MIC_pred_, Predictive modelling method of the minimal important change (MIC); QuickDASH, shortened version of the Disabilities of the Arm, Shoulder and Hand questionnaire.

The MIC_ROC_ for the QuickDASH was 13.6 with a sensitivity of 0.59 and specificity of 0.82, resulting in a change of 36.4% of the baseline score. The MIC_pred_ for the QuickDASH was 10.8 (95% CI 4.84–17.10), resulting in a change of 29.0% of the baseline score. The MIC_ROC_ for the PSFS was 2.0, resulting in a change of 29.4% of the baseline score. The sensitivity and specificity were 0.71 and 0.67, respectively. The MIC_pred_ for the PSFS was 1.9 (95% CI 0.71–3.09), resulting in a change of 28.2% of the baseline score. The visual anchor-based MIC distribution is illustrated in Fig. 3 for both instruments.

When calculating the MICs adjusted for baseline scores, the median QuickDASH score was 39. Of the 52 patients with a low QuickDASH baseline score (< 39), 29 were improved and 23 were stable. 54 patients had high QuickDASH baseline score (≥39), 21 were improved and 32 were stable. The MIC_ROC_ for the QuickDASH was 3.4 and 14.3 for patients with low and high baseline scores, respectively. The median PSFS score was 3. Of the 45 patients with a low PSFS baseline score (< 3), 17 were improved and 28 were stable. 56 patients had high PSFS baseline score (≥3), 31 were improved and 25 were stable. The MIC_ROC_ for the PSFS was 0.8 and 4.0 for patients with low and high baseline scores, respectively.

## Discussion

The results of our study demonstrated that the Norwegian versions of the QuickDASH and PSFS both showed to be responsive when used in patients with shoulder pain referred to an outpatient hospital clinic. The instruments were able to discriminate between improved and non-improved patients as the AUCs were 0.75. Most of the 9 predefined hypotheses were also confirmed. Therefore, we concluded that the QuickDASH and PSFS demonstrated acceptable responsiveness in our population. The MIC values for the total sample ranged from 10.8 to 13.6 for QuickDASH and from 1.9 to 2.0 for PSFS, depending on the method used. Moreover, this study also showed that the MIC values varied according to the baseline scores, which is important to take into account when interpreting MICs in individual patients. To our knowledge, this is the first study to use two anchor-based methods, determined by ROC analysis and by predictive modelling, to calculate the MICs for both PSFS and QuickDASH.

The AUC values in our study for the QuickDASH and PSFS were both 0.75 with overlapping confidence intervals, which indicates that these instruments are equally responsive. For the PSFS, the AUC in our study is somewhat higher than what has been reported in two previous studies on subjects with shoulder disorders undergoing physical therapy, which showed AUC values of 0.67 and 0.71 [5, 35]. For the QuickDASH, four previous studies have reported AUC values, ranging from 0.78 to 0.85, which is slightly higher than the result in our study [11, 30, 35, 55]. The minor differences might be due to differences in the shoulder samples and different follow-up periods (ranging from 4 weeks to 6 months). In another study of responsiveness of the QuickDASH [31], only effect size and standardized response mean were reported, which the COSMIN group defines as inappropriate methods for evaluating responsiveness [31].

A plausible explanation for the somewhat lower AUC for the QuickDASH may be that the GROC was related to change on the most important PSFS item and not to the overall change in shoulder complaints at follow-up. The use of a GROC as an external anchor has been criticised for its reliability and possible object to recall bias [19]. The GROC in this study was construct-specific: with a question formulated in such a way that it should capture a change in activity limitation related to the most important activity in the PSFS. It should therefore be related to disability and the construct of both the QuickDASH and PSFS. This is somewhat reflected in the observed correlations between the anchor and the change scores of the QuickDASH and PSFS, which were moderate (0.47 and 0.50), as we expected (Hypothesis no. 1). A reason why the correlation between the PROMs and the GROC is not higher might be recall bias: patients have difficulty recalling their change in function when the time interval is 3-months [56]. However, a construct-specific GROC used in this study has shown to be more valid compared to generic GROC [57, 58]. Regarding the adequateness of the GROC as an anchor, the observed correlations between the GROC and the QuickDASH and PSFS in this study are higher than the recommendation of Revicki et al. (value > 0.30) [19] and proximate to the recommendation of de Vet et al. (value > 0.50) [24]. Nevertheless, we cannot be sure if the AUC would have been higher with the use of a different anchor.

The MIC values for the QuickDASH in the present study are comparable with previous studies in this population (range, 8.2–13.6) [30–32, 55], whereas the MICs for the PSFS were slightly larger in the present study compared to previous findings in upper extremity patients (range, 1.1–1.3) [5, 36]. One explanation for this difference might be related to whether they used an adequate anchor or not. Koehorst et al. [5] reported a correlation between the GROC and the change scores of PSFS to 0.32, which might indicate that the anchor was not sufficient. Hefford et al. [36] did not report on the correlation, and therefore, it is difficult to know if the GROC was an acceptable anchor. Importantly, when PSFS is used in a range of musculoskeletal conditions other than upper extremity disorders, the MIC (range, 1.3–3.0) is found to be comparable to our results [59–62].

In the present study, we used two different anchor-based methods for the MIC estimation (MIC_ROC_ and MIC_pred_). Since existing literature for the QuickDASH and PSFS mainly has reported MIC_ROC_, this method was implemented for comparison purposes. When evaluating individual patients’ improvement, we propose that the MIC_pred_ values presented in this study are used because of its greater precision compared to the MIC_ROC_ [23]. However, since MIC values are considered method- and context-specific, all available MIC estimates and ranges should be considered when applied to a certain clinical or research context [19, 22, 63]. Additionally, although we used anchor-based methods as proposed by the COSMIN-group, these approaches have been criticised for its risk of recall bias when estimating the MIC value [63]. However, a recent study by Terluin et al. [23] highlights that when the improved and unchanged groups are equally sized the risk of bias will be far less than if the groups were to be skewed. Since the proportion of improved were 0.5 in the present study, we therefore do not consider recall bias a significant weakness in our study.

To determine if a change score is clinically important, the MIC values should be interpreted in relation to the smallest detectable change (SDC) which is closely related to measurement error [20]. Ideally, the SDC should be smaller than the MIC to be 95% confident that the change in an individual patient is statistically significant and is not due to measurement error. We found that the MICs for the QuickDASH (range, 10.8–13.6) did not exceed the SDC of 16.5 reported by Budtz et al. [55] in a comparable sample in patients with shoulder pain. Therefore, the MICs for the QuickDASH in the present study cannot be distinguished from measurement error in individual patients. Regarding the PSFS, the SDC was previously estimated as 0.97 reported by Koehorst et al. [5] in shoulder patients with similar baseline characteristics as in the present study. Based on this SDC, there is 95% certainty that a change of 1.9 was not due to measurement error in individual patients. However, both these SDC values are from different populations and should therefore be interpreted with caution since MIC values vary across different contexts [19, 22].

Consistent with previous literature on MIC estimation, the MICs varied according to the baseline scores [19, 24, 64, 65]. Our results showed that higher baseline scores resulted in higher MIC values. This means that patients with moderate to severe disability need a larger improvement to define this change as important. Thus, we recommend that different MIC values should be used for patients with low or high baseline severity.

### Strengths and limitations

The main strength of this study is that we investigated responsiveness and MIC by using consensus-based methods according to the COSMIN recommendations. This current study contributes to the evidence regarding measurement properties of both the QuickDASH and PSFS among patients with shoulder pain. Another strength is that we adjusted the MICs for baseline scores and included a relatively new method for estimating the MIC, the predictive modelling of MIC, which has been found to be a more accurate calculation of the anchor-based MIC [23, 25]. Instead of reporting a single fixed value, these different MIC values can promote a more accurate interpretation of both the PROMs change scores.

The main limitation of the present study is the relatively small sample size in the subgroup analysis when estimating the MIC according to baseline severity. Moreover, although we found AUC values above the 0.70 level of acceptable responsiveness, the lower borders of the confidence intervals were just below 0.70 for both the QuickDASH (0.66) and PSFS (0.65). This should be taken into account when interpreting these estimates. Another limitation of this study is the lack of opportunity to estimate the SDCs of the PROMs, since only two time-points were assessed. Also, the patients were predominantly female, thus affecting the generalisability to other populations. ﻿Despite these limitations, our results generalise to patients with shoulder pain who are likely to be encountered in a hospital-based outpatient clinic. ﻿However, further responsiveness studies in more general contexts are recommended.

## Conclusions

Based on the COSMIN standards, the Norwegian versions of the QuickDASH and PSFS are responsive and able to capture change in disability. Both instruments are similarly able to discriminate between patients that have improved and patients that are unchanged. The MIC values for both the questionnaires varied based on baseline score and method used. We recommend taking these MIC values into account when measuring improvement or planning clinical studies on a similar sample.

## Data Availability

The data that support the findings of this study are not publicly available due to personal data protection. An anonymous form of the data can be made available on request from the corresponding author, TR.

## Abbreviations

AUC: Area under the receiver operating characteristic curve
CI: Confidence interval
ES: Effect size
GROC: Global rating of change scale
HSCL-25: Hopkins Symptom Checklist
MIC: Minimal important change
MIC_ROC_: Receiver operating curve method of the minimal important change
MIC_pred_: Predictive modelling method of the minimal important change
NRS: Numeric pain rating scale
PSFS: Patient-Specific Functional Scale
QuickDASH: Short form of the Disabilities of the Arm, Shoulder and Hand questionnaire
ROC: Receiver Operating Curve
SDC: Smallest detectable change
SQK: Single substitute question for kinesiophobia
SRM: Standardised response mean
WA: Workability
